# Field Evaluation of Spent *Pleurotus ostreatus* Substrate Reveals Limited Suppression of Fusarium Wilt in Banana

**DOI:** 10.3390/jof11110816

**Published:** 2025-11-18

**Authors:** Walter Ocimati, Geofrey Ogwal, Elizabeth Kearsley, Guy Blomme

**Affiliations:** 1 Bioversity International, Kampala P.O. Box 24384, Uganda; ogwalgeff1996@gmail.com; 2 BlueGreen Labs, 9120 Melsele, Belgium; elizabeth@bluegreenlabs.org; 3 Bioversity International, c/o International Livestock Research Institute, Addis Ababa P.O. Box 5689, Ethiopia; g.blomme@cgiar.org

**Keywords:** farmyard manure, Foc R1, *Musa* spp., soil amendments, SPoS

## Abstract

*Fusarium oxysporum* f. sp. *cubense* (Foc), the causal agent of Fusarium wilt of banana, can persist in the soil for extended periods as chlamydospores or endophytes in weeds, complicating control measures. No single control strategy is effective. Biological agents present an increasingly important control option. This study explored the potential of the spent *P. ostreatus* substrates (SPoS) to suppress Foc R1 in a field with high Foc inoculum, following laboratory and greenhouse studies that highlighted the potential of *P. ostreatus* as a biocontrol agent against Foc. A susceptible cultivar ‘Sukali Ndizi’ and a resistant cultivar ‘Mpologoma’ were used for the study. SPoS was compared with farmyard manure (FYM), a combination of SPoS with FYM and a control without treatment. A one-time application of the treatments at planting did not consistently and significantly (*p* > 0.05) reduce the prevalence and severity of leaf symptoms, pseudostem splitting and corm damage in the mother and ratoon plants of the susceptible cultivar. No symptoms occurred in ‘Mpologoma’. SPoS applications at planting and after every two months over an 8-month period did not significantly reduce leaf symptoms and corm damage in ‘Sukali Ndizi’, while it increased pseudostem splitting. The marginal and irregular reductions in FW could be due to an observed high weevil damage in SPoS treatments and other confounding factors such as weather, SPoS quality, and pathogen load in the field. Further research on weevil–SPoS interactions, use of *P. ostreatus* mycelium-rich substrate, and other confounding factors is crucial for fine tuning *P. ostreatus* use.

## 1. Introduction

*Fusarium oxysporum* f. sp. *cubense* (*Foc*) is a soil-borne fungus responsible for Fusarium wilt (FW), a devastating disease affecting banana plants globally [1]. *Foc* is classified into three main races (Race (R) 1, R2 and R4) based on their pathogenicity towards different banana cultivars. Race 1 primarily attacks varieties such as Gros Michel, Silk, Pome and Pisang Awak [2,3]. Race 2 primarily affects Bluggoe (ABB) and closely related cooking bananas, while R4 affects the Cavendish group and a good portion of the R1 and R2 susceptible varieties [4].

The soil-borne pathogen colonizes banana xylem vessels in roots, corms, leaf sheaths and leaves, disrupting water and nutrient transportation, resulting in external symptoms such as leaf lamina chlorosis, leaf yellowing and wilting, and ultimately plant death. FW poses a serious threat to commercial banana production and food security, affecting approximately 400 million people who depend on bananas as a staple food or source of income globally [5]. The disease can cause up to 100% yield loss and affects the sustainable use of banana production landscapes [5]. This pathogen can persist in the soil for extended periods as chlamydospores or as endophytes in various weed species, complicating control measures and leading to substantial yield losses [6]. To date there is no single fully effective strategy for managing the disease across affected cultivars. Integrated disease management strategies, incorporating the use of resistant varieties and improved agricultural practices, are essential to mitigate the impact of Foc on banana production systems. The use of suppressive biological agents in the management of Foc presents an increasingly important area of research. This study specifically explored the potential of the residual remains of *Pleurotus ostreatus* in spent *P. ostreatus* substrates (SPoSs) to suppress Foc R1 under field conditions.

Recent studies have highlighted the potential of *P. ostreatus* not only as a valuable edible mushroom but also as a biocontrol agent against plant pathogens. *P. ostreatus* and the spent substrate derived from its cultivation have been shown to possess inhibitory effects on the growth of multiple plant pathogenic organisms including Foc [7,8,9,10,11,12]. This suggests that its substrate waste could be used in integrated disease management strategies. Specifically, experiments conducted under controlled laboratory and greenhouse conditions have demonstrated that the mycelium of *P. ostreatus* can suppress Foc through mechanisms such as competition for resources, antibiosis, and possibly predation [10]. Though *P. ostreatus* is not known to live as an endophyte in plant tissues unlike well-known biocontrol agents [e.g., *Bacillus* spp. and *Trichoderma* spp.], it is thus hypothesized that these mechanisms could reduce Foc inoculum in the soil, thus minimizing infections.

In laboratories and greenhouses, conditions are often optimized, simple and tightly regulated for the performance of biocontrol agents. Biocontrol agents are often applied in high doses, facilitating their establishment. However, field conditions are heterogenous with several complicated interactions due to environmental-, biological- (e.g. varying soil microbiomes, plant species), physiochemical- and management-related factors, often leading to failure of biocontrol agents under field conditions [13,14]. For example, they can be outcompeted by the diverse native microbiome acting as antagonists or pathogens and be affected by soil dynamics in the field [15,16,17,18]. Under field conditions, the efficacy of a biocontrol agent can also be limited by high or variable pathogen pressure and coexistence of multiple pathogens [19]. Fravel [14] also reports inconsistent formulation of microbial biocontrol agents to be a major challenge limiting their survival, ease of use, and field efficacy. These factors affect the ability of biocontrol agents to persist and colonize the plant’s rhizosphere. Thus, mixed outcomes have been realized under field conditions. For example, Wang et al. [20] showed banana–pineapple rotation coupled with a biofertilizer application to lead to suppression of banana Fusarium wilt, with an increase in the *Burkholderia* genus. In contrast, Bubici et al. [21] reported a mixed outcome with several biocontrol agents including *Pseudomonas* and *Trichoderma* strains to achieve moderate to high (70–79%) efficacy against Foc under field conditions, while others like *Bacillus*, arbuscular mycorrhizal fungi and nonpathogenic Fusarium strains gave lower (42–55%) efficacy. Thus, the dynamics of interactions in the field are rarely replicable in controlled laboratory or screenhouse environments, making it difficult to predict how biocontrol agents will perform in real field ecosystems [16]. Understanding these field interactions is crucial for strategies in designing more effective biocontrol agents. It was with this in mind that it was crucial to evaluate SPoSs under naturally Foc-infested soil conditions to assess its efficacy to inform the next steps in the development of the innovation. This research thus assessed the effect of SPoS on FW disease in banana under field conditions.

## 2. Materials and Methods

Two field experiments evaluated the effect of the spent substrate of *P. ostreatus* on FW leaf symptom prevalence per visit and severity using tissue culture-derived banana plantlets of a Foc R1 susceptible cultivar in a field infested heavily with Foc at the National Agricultural Research Laboratories in Kawanda, Uganda. The field had previously been artificially inoculated to screen banana cultivars and hybrids against Foc R1. At onset of the *P. ostreatus* experiments, soil-level Foc R1 inoculum load was hence substantial. The field was highly heterogenous with soil pH, soil nitrogen (N), potassium (K), and phosphorus, respectively, varying between 4.82 and 5.84, 13.2 and 85 mg/L, 145 and 310 mg/L, and 9 mg/L and 48.5 mg/L. The soils, SPoS and FYM were tested using the SKW 500 (Palintest) Complete Soil Kit [22].

### 2.1. Experiment 1

In the first field experiment, the effect of a single SPoS in the soil application on FW development was assessed. Here, four treatments were assessed, namely (i) application of SPoS only, (ii) application of decomposed cow dung manure (Farmyard manure; FYM) only, (iii) application of a combination of SPoS with FYM, and (iv) a control without SPoS and/or FYM. SPoS was obtained from a single commercial farm in Wakiso district, central Uganda. The SPoS was already highly decomposed at time of application and some of the rearing units (gardens) had visible actively growing *P. ostreatus* mycelia while others did not. The choice of using SPoS instead of fresh and actively growing mycelia was a practical compromise for scalability of the innovation. Mushroom production can be integrated on banana farms using banana and household waste as substrate and the spent waste after mushroom harvest applied on farm for managing soil and plant health. The SPoS sourced from different rearing units were crushed and thoroughly mixed to minimize potential variations that would arise from differences between the rearing units. The crushing also eased SPoS application. The SPoS had a pH of 7.72, salt content of 867 ppm, and EC of 1733 uS. Its N, K, and P contents were, respectively, 17 mg/L, 455 mg/L, and 22 mg/L. For the SPoS-only treatment, three spades of SPoS (~2.6 kg, air dried weight) were added to each planting hole and mixed with the topsoil to form a mix with 30% SPoS (*v*/*v*). This mimicked soil–SPoS ratios used in the pot trials in Ocimati et al. [10]. Tissue culture-derived banana plantlet roots were directly inserted into the SPoS–top soil mixture. For the decomposed cow dung manure treatment, three full spades (~2.6 kg, air dried weight) were applied and mixed with the topsoil per banana planting hole. The FYM had a pH, salt content, EC, N, K and P of, respectively, 8.81, 463 ppm, 944 uS, 1.9 mg/L, 455 mg/L and 84 mg/L. For the FYM treatment, 3 spades of FYM were mixed with the topsoil, while for the SPoS + FYM treatment, three spades each of SPoS and FYM were mixed with the topsoil before planting the banana plantlet. For the control treatment, the topsoil was put back into the planting hole, and the banana plantlet roots were embedded within the topsoil.

Two banana cultivars, (i) a Foc R1 susceptible cultivar, Sukali Ndizi’ (Apple banana, *Musa* AAB genome) and (ii) a Foc R1 resistant cultivar ‘Mpologoma’ (*Musa* AAA-EAH genome) as a control were established in the field experiment. The tissue culture-derived plantlets were obtained from a certified private commercial banana multiplier. A randomized complete block design (RCBD) with four blocks and 32 plots was used. The field was split into blocks (measuring 90 m by 11 m). Each block was split into 8 plots measuring 9 × 11 m. Gaps of 3 m and 2 m were left between the blocks and the plots, respectively. Within each block, the two banana cultivars and thereafter the four soil amendment treatments were randomly assigned to minimize variations within the blocks arising from the slope gradient (~4%) and a possible variation in soil inoculum load. This resulted in two plots per treatment type per block and 1 treatment plot per cultivar per block, replicated in 4 blocks. A total of 20 plants (4 rows × 5 columns) were established in each treatment plot using a spacing of 2 × 2 m. A guard row of the EAHB cultivar ‘Mbwazirume’ was planted 3 m away from the blocks. The experiment was established on the 9 November 2022.

The fields were monitored for Foc symptom development during the plant crop and first ratoon crop (i.e., second cropping cycle). No de-suckering or de-trashing (i.e., the removal of old leaves) was applied to minimize the risk of introducing and spreading Xanthomonas wilt disease that was present in the neighboring fields. Minimum tillage by hand hoeing was carried out to control the weeds.

Data on FW aboveground symptom prevalence and severity were collected at least monthly, from 7 months onwards to 2 years after experiment establishment, on the mother plant and ratoon crops (i.e., lateral suckers). As such, data collection was initiated when first symptoms were observed. At each interval, symptom severity scores were determined for each mother plant in the plots, while an average symptom severity score for all attached suckers in a mat was computed for 3 mats per plot. Thus, *n* of 80 plants/mats per treatment nested within four plots for mother plants and *n* of 12 plants/mats nested within four plots for the ratoon crops were used. A modification of the scale of 1 to 5 [23] to a score of 0 to 4 was used for leaf symptom severity assessment. The scores of 0, 1, 2, 3 and 4, respectively, represented no visual leaf symptoms, 1–33% of predominantly older leaves turning yellow, 34–66% of leaves turning yellow with some older/outer leaves hanging down the pseudostem, 67–100% of leaves turning yellow with some outer leaves hanging down the pseudostem, and plant death with multiple brown leaves hanging down the pseudostem. Pseudostem splitting was ranked on a score of 0 to 2, with 0, 1 and 2 denoting, no pseudostem splitting, mild splitting and severe splitting, respectively. The pseudostem splitting score was a modification of the 1 to 3 score used by Buryegyeya et al. [24,25]. Leaf and pseudostem symptom severity scores were assessed by one trained field assistant to ensure consistency throughout the experiment.

Corm damage was only assessed in the 1st ratoon crop at the termination of the experiment (i.e., 24 months after experiment initiation). Three plants in three randomly selected banana mats per plot (i.e., *n* = 12 plants/mats per treatment nested within 4 plots) were destructively sampled and assessed using a scale of 0 to 4 (amended from Viljoen et al. [23]). The scores from 0 to 4, respectively, denote no internal corm symptoms, few internal spots to 1/3 discolored, 1/3–2/3 discolored, >2/3 discolored, and all inner corm sections (cortex to central cylinder) discolored.

Growth and yield data were collected for the mother plants of both cultivars. The parameters assessed included the time from planting to flower emergence and the time from planting to harvest of the physiologically mature bunch. Upon harvest, the number of clusters (i.e., hands) and bunch weight were also determined.

### 2.2. Experiment 2

A second field experiment was established to ascertain the effect of multiple SPoS applications on FW development in the Foc R1 susceptible ‘Sukali Ndizi’ plants in the same Foc-affected field used for experiment 1. The experiment was established on 31 January 2024. Two plots containing a total of 12 plants each (arranged in 3 lines of 4 plants) were established, with one plot treated with SPoS at planting as in experiment 1 and the other left untreated as a control. A simple randomized controlled experiment was used for the experiment. In the SPoS treatment plot, the first SPoS treatment application was carried out as for experiment 1. This was followed by additional aboveground applications at 2-, 4-, and 6-month post-experiment initiation, each consisting of three spades of SPoS applied on the soil surface in a 30 cm wide ring around the base of the plants. Data were collected on FW prevalence per visit, including leaf and pseudostem symptom severity as in experiment 1. Eight months after planting, the plants were destructively sampled to score for FW corm damage as described in experiment 1.

### 2.3. Data Analysis

An analysis of variance was conducted to determine the effects of the treatments on FW prevalence per visit and symptom severity development, as well as on growth and yield metrics. The Tukey test at 5% probability level was used for mean separation. Data analysis was performed using R Statistical software version 4.4.0 [26].

## 3. Results

### 3.1. Experiment 1

**Figure 1:**
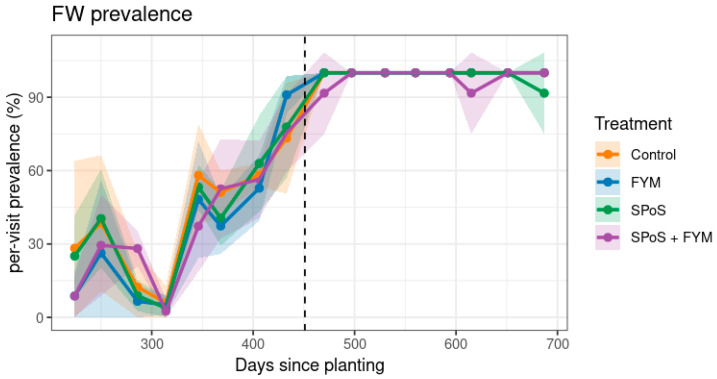
Fusarium wilt prevalence of the susceptible cultivar ‘Sukali Ndizi’ as percentage of plants per plot developing initial (leaf yellowing) symptoms throughout experiment 1. Shaded bands show SD.

The susceptible cultivar ‘Sukali Ndizi’ was affected by FW in all treatment and control plots, and FW leaf symptom prevalence was similar across all treatments and control (Figure 1). By day 250 since planting, FW prevalence ranged from 26–41% across all plots, but a notable recovery occurred by day 314, reducing leaf symptom prevalence to 2–6% across all plots, including controls. The recovery period coincided with the main dry months of June to August (Figure S1). Despite the recovery, FW prevalence steadily rose until day 433, the final day of monitoring mother plants, reaching between 66.2 ± 10.9% in the control and 83.8 ± 3.8% in SPoS + FYM, with no significant differences between treatments and controls (Figure 1). In the ratoon crop, FW prevalence reached 100% across all treatments and controls. The resistant cultivar ‘‘Mpologoma’’ remained asymptomatic for FW throughout the experiment, showing no external or internal symptoms in either treatment or control plots.

Fusarium wilt leaf symptom severity on the final day of monitoring the mother plants of the susceptible cultivar ‘Sukali Ndizi’, i.e., 433 days after planting and treatment application, only differed significantly (*p* = 0.0182) for plants that died due to FW (i.e., symptom score 4) (Table 1). Significantly more (*p* = 0.0182) plants were dead in the control (7.4 ± 1.0%) compared to 0.0% in the SPoS group. However, the control did not differ from the FYM (7.8 ± 2.7) and SPoS + FYM (2.6 ± 2.6) treatment (at 5% Tukey HSD test) in the number of dead plants due to FW disease. Approximately 9.0 ± 3.8% (in FYM) to 26.7 ± 11.3% (in control) of plants had no visible leaf symptoms (i.e., score 0) at 433 days. Though non-significant, the treatments with SPoS had more plants (71% to 74%) with no symptoms (i.e., score ‘0’) and about 1–33% of leaves yellowing (i.e. score ‘1’) due to FW (Table 1). Higher overall FW leaf symptom severity scores than in the mother plants were observed in the ratoon crop assessed on day 687 (i.e., final day of monitoring). The ratoon crop had no significant differences (*p* > 0.05) between the treatments in leaf symptom severity score thresholds 0 (no symptoms) to score 3 (67–100% leaves turning yellow). A significantly higher (*p* = 0.001) number (33.3%) of dead plants (score 4) were observed in the control treatment (Table 1).

Pseudostem symptom splitting score distributions on the mother and ratoon plants at 433 and 687 days, respectively, did not differ significantly (*p* > 0.05) between the treatments at the three pseudostem splitting score scales/thresholds between the treatments (Table 2).

Corm damage scores assessed only in the ratoon crops at 687 days did not differ significantly (*p* > 0.05) between the treatments for the five corm damage score thresholds (i.e., 0–4) (Figure S2). Most plants (60 to 80%) had a few internal spots to two-thirds of the corm discolored due to FW infection.

**Figure 2:**
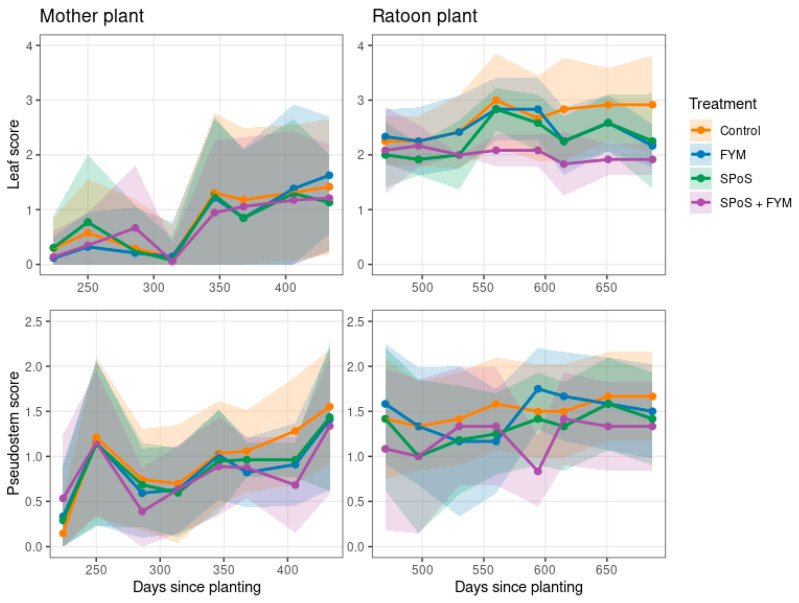
Fusarium wilt symptom development of the susceptible cultivar ‘Sukali Ndizi’ throughout experiment 1. Leaf scores (top row) and pseudostem scores (bottom row) for mother plants (first column) and the ratoon crop (second column), recorded at time of visit.

The mean symptom severity varied inconsistently across treatments, with many monitoring intervals showing no statistically significant differences (*p* > 0.05) (Figure 2; Table S1). Specifically, mother plant leaf symptoms remained similar to the control throughout the experiment in all soil amendment treatments (Table S1). However, the development of pseudostem symptom severity showed more variation. The SPoS + FYM treatment exhibited significantly lower pseudostem symptom severity compared to the control on days 286, 368 and 406, though it showed increased severity on day 224. Additionally, FYM and SPoS treatments also demonstrated significantly lower pseudostem severity on days 368 and 406 (Table S1).

In the ratoon crop, mean leaf symptom severity was lower in the SPoS + FYM treatment from day 560 onward (Figure 2, Table S2). From day 600, leaf symptom severity was also lower in the SPoS and manure treatments. The SPoS and SPoS + FYM treatments had lower pseudostem symptom severity scores in the control, though for SPoS + FYM was only significantly lower (*p* < 0.05) than the control on day 594.

No significant difference was observed in the time needed for each cultivar to reach the flowering stage across treatments (Table 3). Few (0–6) Sukali Ndizi’ plants developed mature bunches, with none in the SPoS treatment. Furthermore, no significant difference was observed among the yield parameters of the few ‘Sukali Ndizi’ plants that produced harvestable bunches. Harvest parameters for the ‘Mpologoma’ cultivar were only assessed for 26–51% of plants across the treatments. This low assessment rate was due to the loss of mature bunches from theft, as well as plant snapping and toppling due to pest damage, and wind damage during the bunch filling stages. Plant snapping and toppling were observed to be mainly caused by weevil and nematode infestation, respectively, although the pest burden was not quantified. The number of days from planting to harvest of ‘Mpologoma’ was significantly longer in the FYM treatment than in the SPoS and control treatments (617 ± 42 days since planting, *p* < 0.01), although no significant impact was observed in yield parameters. Critically, SPoS amendments had no observed effect on either growth or yield parameters.

### 3.2. Experiment 2

In experiment 2, leaf symptom incidence and severity with time (i.e., from 168 to 238 days), showed no significant difference (*p* > 0.05) in the number of plants for each severity threshold (measured on a scale 0 to 4) between SPoS and the control treatment (Table 4). The number of plants without FW-characteristic leaf yellowing symptoms over time varied between 92% and 56% for SPoS compared to 83% and 50% for the control treatment. No plants reached 67% leaf yellowing (score 3) or died (score 4) due to FW, though more control plants (17%) had 34–66% of their leaves affected compared to none in the SPoS treatment (Table 4). However, by the end of the experiment, five plants (three and two plants in the SPoS and control treatments, respectively) had died, whereas others had their corms highly damaged by weevil larvae (*Cosmopolites sordidus*). Weevil damage was observed to be more severe in the SPoS treated plants.

Across the two treatments, pseudostem splitting was only observed from day 202, with 100% of plants under SPoS compared to none under control having mild pseudostem split (Table 5). Higher scores for pseudostem splits in SPoS compared to the control were observed from day 202 to 238 across all days of assessment, with significant differences (*p* < 0.05) observed on days 202 and 218. On day 238, 61% of plants in the SPoS treatment had severe pseudostem split, whereas all plants in the control had recovered.

**Figure 3:**
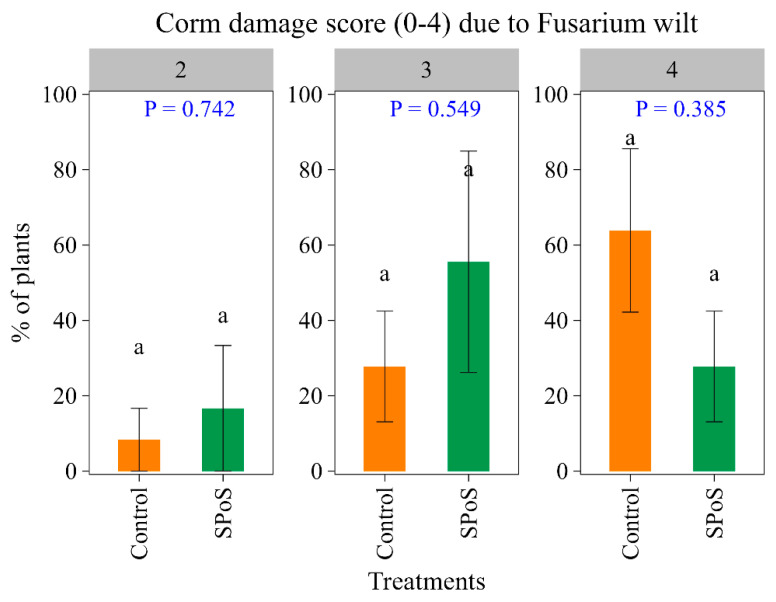
Percentage of plants treated with spent *P. ostreatus* substrate (SPoS) or without SPoS (control) expressing different corm damage score thresholds of 0 to 4, 230 days after treatment application. Corm damage scores of 0 to 4, respectively, denote no internal corm symptoms, few internal spots to 1/3 discolored, 1/3–2/3 discolored, >2/3 discolored, and all inner corm sections (cortex to central cylinder) discolored. Means followed by the same letter are not significantly different at 5% Tukey HSD.

The impact of the treatments did not differ significantly on corm damage severity profiles assessed 238 days after trial initiation. Corm damage scores varied from 1/3 of corm discoloration to complete discoloration of corm tissues. However, though non-significant (*p* = 0.385), a lower proportion of SPoS-treated plants compared to control had their corms completely discolored due to the infection (i.e., score 4; Figure 3).

## 4. Discussion and Conclusions

A two-year field experiment evaluating a single application of SPoS and/or FYM on the Foc R1-susceptible ‘‘Sukali Ndizi’’ cultivar revealed limited and inconsistent reductions in FW symptom severity in both mother plants and ratoon crops. These reductions did not translate into improved bunch development or reduced FW prevalence compared to untreated controls. Furthermore, SPoS alone or in combination with FYM yielded only sporadic and minor symptom severity improvements. With repeated SPoS applications in a separate experiment, leaf yellowing, pseudostem splitting and corm damage symptoms due to FW severity were significantly different or reduced. These inconsistent results can be attributed to the multiple confounding factors in the field, suggesting the need for further studies to disentangle these factors to inform further development of the innovation as a component of integrated FW management.

These field experiments are inconsistent with results from in vitro and greenhouse potted plant experiments [10,27]. Ocimati et al. [10] identified potential putative competition, antibiosis and predation mechanisms of *P. ostreatus* in the suppression of Foc. In these in vitro experiments, *P. ostreatus* hyphae were observed to enlarge and become more vigorous at points of contact with Foc hyphae, followed by the degeneration of Foc on media. The study also observed significant reductions in corm damage in potted plants due to application of a substrate containing actively growing *P. ostreatus*. Additionally, antifungal activity from *P. ostreatus* and other mushroom substrates has been reported across a range of *Fusarium oxysporum* formae speciales, including those affecting tomato, melon and shallot [8,27,28]. This antagonism, attributed to the diverse microbial community and secondary metabolite production within the spent mushroom substrate [7,27,28,29], may provide substantial control against pathogenic fungi. The discrepancies between the field experiments in this study and in vitro/greenhouse experiments suggest a complexity that may not be fully captured in controlled settings and underscores the challenges of translating controlled environment results to real-world conditions. Failures of potential other bio-control agents under field conditions despite clear successes in vitro and in pot experiments have been reported before [30,31]. The failures are generally attributed to variations in the field, the weak ecological competence of the bio-agents, the microbe inherent traits, pathogen load and quality of the bio-agent carrier substrate [31,32,33]. Further research is crucial to elucidate the interaction of *P. ostreatus* with the diverse environmental variables and the pathogens in field conditions.

Although this study does not identify the specific field factors limiting the efficacy of SPoS as a biocontrol agent against Foc under field conditions, several potential constraints can be inferred. Firstly, the quality of the SPoS might have been insufficient or variable. Spent mushroom waste was sourced from gardens (*P. ostreatus* rearing unit) which were partially decomposed and often dry. Some of the gardens did not have visible growing mycelia. The potency of SPoS is influenced by the size of actively growing mycelia, and the decomposed SPoS likely had less of the actively growing agents and was thus less effective in suppression of Foc. The dry waste substrate could also have had a decreased biodiversity of microorganisms which are critical for their antagonism toward pathogenic fungi [27,28]. It is also important to note that the study in Ocimati et al. [10] used a substrate with actively growing *P. ostreatus* mycelia. The use of actively growing *P. ostreatus* mycelia possibly on millet or sorghum grains or other organic carrier material could be explored, though is likely not economical for large-scale applications. Sourcing high quality SPoS by smallholders would likely depend on on-farm mushroom cultivation, with farmers growing the mushrooms on banana organic waste [e.g. chopped up leaves and pseudostems], after which the used substrate could return to their banana fields. This could further provide nutrition, income and soil health benefits. However, this method would likely yield a highly variable product and outcome. Given the inconsistent and even discouraging results from SPoS in this study, the appropriate timing and management of SPoS on-farm would need to be studied and optimized.

Secondly, given that the roots of the banana plants were embedded in the substrate, its chemical composition could have potential agronomic and phytopathological implications for the banana crop. The SPoS had a slightly alkaline pH (7.72), creating less-favorable conditions for Foc proliferation, given higher soil pH levels have been associated with reduced Fusarium wilt severity in bananas [34,35]. Its relatively high potassium (455 mg L^−1^) and moderate phosphorus (22 mg L^−1^) concentrations are also beneficial for improving banana vigor and resistance. Optimal K and P nutrition have been shown to enhance plants’ structural integrity and defense mechanisms against vascular pathogens [35,36]. The N content was low (17 mg L^−1^) which would be advantageous, as excessive N predisposes banana to Fusarium wilt [34]. Nevertheless, the electrical conductivity (1.73 dS m^−1^) and salt content (867 ppm) suggest moderate salinity that could have induced physiological stress, potentially increasing susceptibility to Foc. High salinity has been reported to exacerbate Foc in banana [37] and *Fusarium oxysporum* of other crops [38,39]. Therefore, the amount of SPoS rate applied and the timing of introducing the plants needs to be carefully examined to minimize potential salinity risks in the root zone. Possibly, plantlets could be introduced after several rainfall events that would drain part of the salts.

Thirdly, the high soil-level fungal load in the study site could have also overwhelmed the biocontrol agent, reducing its effectiveness. A site previously inoculated with Foc R1 and cultivated with a susceptible banana cultivar was used for the study. High pathogen load has been reported to result in an incomplete suppression by biocontrol agents [33,40]. For example, a very high efficacy of *Bacillus subtilis* QS713 was reported by Bardin et al. [41] against *Botrytis cinera* at the recommended dosage of the biocontrol agent, whereas a 10-fold dilution resulted in diverse responses.

Fourthly, direct contact between the residual *P. ostreatus* and *Foc* mycelia may have induced chlamydospore formation, as observed in vitro [10]. This likely represents a defense mechanism by *Foc* to survive in the presence of *P. ostreatus*. These chlamydospores can subsequently germinate in the absence of active *P. ostreatus* mycelia, potentially leading to a rapid increase in the disease.

Multiple studies have shown that composts rich in organic matter and favorable microbial communities contribute to pathogen suppression by increasing soil microbial antagonism, producing toxic volatile organic compounds, inducing systemic resistance in plants, and improving overall soil health and plant vigor [42,43]. Similar effects anticipated from the application of SPoS could have been undercut by a possible positive interaction between SPoS and banana weevils. Experiment 2 revealed a higher banana weevil (*Cosmopolites sordidus*) infestation in plants treated with SPoS. Mulch and organic materials create suitable damp habitats that attract and increase weevil population and damage [44]. The repeated application of SPoS every two months could have thus created suitable, damp micro-environment for the weevils to hide and multiply. Consequently, the damage caused by weevil larvae could have further increased plant susceptibility to *Foc infection*. Even without such amendments, banana weevils show a preference for Foc-infected plant tissues [45] and are a vector contributing to the dissemination of Foc [46,47]. If SPoS amendments further create suitable conditions for weevils, its application will only aggravate the *Foc* problem if no efforts are made to control weevils altogether. Furthermore, no yield improvements were associated with either SPoS or SPoS + FYM amendments, even in the *Foc* R1-resistant cultivar ‘Mpologoma’. It is possible that any expected growth and yield benefits from these organic amendments may have been negated by heightened corm damage attributed to weevil activity. The use of SPoS may thus need to be accompanied with weevil control. The interaction of SPoS amendments with weevils and FW development under field conditions thus needs to be further studied.

*Pleurotus ostreatus* also produces chitosan [48,49], a biopolymer derived from chitin, which induces plant defense responses by enhancing chitinase activity and activating phenylpropanoid pathways, leading to increased resistance against pathogens, including fungi [49,50]. It has also been shown to directly inhibit fungal growth and spore germination through its cationic nature, which disrupts microbial membranes. These benefits could have also been undercut by the multiple interacting factors in the field, including the banana weevil effect.

Fifthly, *P. ostreatus* can only survive on dead organic matter. It lacks endophytic properties and does not colonize the banana rhizosphere [51]. Any effect on the pathogen at the root and rhizosphere level would therefore rely solely on metabolites released from the spent substrate and leached towards the banana roots and possibly active predation on/competition with Foc mycelia in the soil. The banana root system is mainly found in the upper 50 cm soil layer and can extend up to 3 m from the banana stem base [52]. This vast network of roots greatly limits the potential impact of *P. ostreatus* metabolites on *Foc* once the pathogen has invaded the banana root system and corm. Even a highly effective biocontrol agent may need to colonize not only the root and corm tissues but also the pseudostem and leaf tissues, or exert a systemic effect throughout the entire plant, in order to ward off pathogen infection/entry at the whole-plant level. The limited impact of *P. ostreatus* Foc R1 could thus have been partly attributed to a lack of endophytic properties to exert a systemic effect.

The environmental conditions, i.e., variations in temperature and rainfall, could have impacted on the interaction between the biocontrol agent, the pathogen and the host plant. This could explain the observed recovery of banana plants by day 314, which coincided with the main 3-month-long dry season, and the subsequent linear increase in disease incidence. Pegg et al. [53] reported Foc to be favored in dry soil conditions, under which it can extract sufficient water for its growth and reproduction with less antagonism or competition with other microbes. Foc incidence has been observed to develop slowly in the dry season and to increase shortly after the onset of the wet season, when conditions were most favorable for plant growth [1,54]. In contrast, the growth of *P. ostreatus* mycelia and its enzymatic activity are dependent on sufficient moisture. Conversely, high moisture increases the susceptibility of banana plants to Foc infections and FW development. Stover [1] and Simmonds [55] reported actively growing plants to favor FW development. Thus, the dry season possibly gave Foc an advantage over *P. ostreatus*. Further field studies under variable moisture regimes are thus essential to optimize the use of *P. ostreatus* as a biocontrol agent.

Ultimately, the few marginal improvements in FW symptom severity observed at irregular intervals due to SPoS, combined with a risk of adverse interactions with banana weevils and formation of chlamydospores at contact sites with SPoS, suggests that more field studies are necessary to finetune the innovation prior to recommending it as a biocontrol agent for Foc. Further research is needed to fully capture the constraints and improvement opportunities of SPoS amendments under field conditions. The impact of *P. ostreatus* on Foc R1 also needs to be examined using a substrate with an active mycelia. Studies are specifically needed to understand the interaction between SPoS and banana weevils.

## Figures and Tables

**Table 1:** Percentage of mother and ratoon banana plants at different severity score levels 433 days and 687 days, respectively, after planting and application of amendments. FYM and SPoS denote farmyard manure and spent *Pleurotus ostreatus* substrate. Controls had no external substrate. For mother plants *n* = 80 mats nested within 4 plots, while 12 mats nested in 4 plots for ratoon crops.

Treatments	* % Mother Plants (±SE) at Different Leaf Symptom Severity Score (0–4) Thresholds on Day 433				
	0	1	2	3	4
Control	26.7 ± 11.3 a	34.0 ± 16.4 a	17.7 ± 6.1 a	14.2 ± 3.7 a	7.4 ± 1.0 a
FYM	9.0 ± 3.8 a	47.2 ± 7.0 a	23.1 ± 9.7 a	13.0 ± 4.6 a	7.8 ± 2.7 a
SPoS	22.2 ± 10.4 a	53.2 ± 9.4 a	14.1 ± 4.2 a	10.5 ± 5.7 a	0.0 ± 0.0 b
SPoS + FYM	24.6 ± 7.9 a	46.8 ± 16.0 a	18.1 ± 4.6 a	7.9 ± 6.3 a	2.6 ± 2.6 ab
F value	1.436	1.086	0.318	1.485	5.703
df	3	3	3	3	3
*p* value	0.2957	0.4033	0.8123	0.2832	0.0182
**Treatments**	* % Ratoon Plants (±SE) at Different Leaf Symptom Severity Score (0–4) Thresholds on Day 687				
	**0**	**1**	**2**	**3**	**4**
Control	0.0 ± 0.0 a	0.0 ± 0.0 a	41.7 ± 8.3 b	25.0 ± 8.3 a	33.3 ± 0.0 a
FYM	0.0 ± 0.0 a	0.0 ± 0.0 a	83.3 ± 9.6 a	16.7 ± 9.6 a	0.0 ± 0.0 b
SPoS	8.3 ± 8.3 a	0.0 ± 0.0 a	50.0 ± 9.6 b	41.7 ± 16.0 a	0.0 ± 0.0 b
SPoS + FYM	0.0 ± 0.0 a	8.3 ± 8.3 a	91.7 ± 8.3 a	0.0 ± 0.0 a	0.0 ± 0.0 b
F value	1.000	1.000	7.800	3.000	2.745 × 10^31^
df	3	3	3	3	3
*p* value	0.4363	0.4363	0.0071	0.0877	<0.001

* Leaf severity scores of 0, 1, 2, 3 and 4, respectively, represent no visual leaf symptoms, 1–33% of leaves turning yellow, 34–66% of leaves turning yellow with some old leaves hanging down the pseudostem, 67–100% of leaves turning yellow with some old leaves hanging down, and plant death with multiple brown leaves hanging down the pseudostem. Means followed by the same letter are not significantly different.

**Table 2:** Percentage of mother and ratoon banana plants at different pseudostem splitting score levels 433 and 687 days, respectively, after planting and application of amendments. FYM and SPoS denote farmyard manure and spent *Pleurotus ostreatus* substrate. Controls had no external substrate. For mother plants *n* = 80 mats nested within 4 plots, while 12 mats nested in 4 plots for ratoon crops.

Treatments	* % Mother Plant (±SE) at Different Pseudostem Splitting Score Thresholds on Day 433		
	0	1	2
Control	7.1 ± 2.4 a	30.2 ± 1.8 a	62.8 ± 1.9 a
FYM	18.1 ± 3.6 a	23.1 ± 3.4 a	58.8 ± 4.6 a
SPoS	20.5 ± 10.1 a	14.6 ± 4.2 a	64.8 ± 8.6 a
SPoS + FYM	17.5 ± 4.2 a	33.5 ± 8.6 a	49.0 ± 12.5 a
F-value	1.135	2.365	0.857
df	3	3	3
*p* value	0.3859	0.1389	0.4974
**Treatments**	* % Ratoon Plants (±SE) at Different Pseudostem Splitting Score Thresholds on Day 687		
	**0**	**1**	**2**
Control	0.0 ± 0.0 a	33.3 ± 13.6 a	66.7 ± 13.6 a
FYM	8.3 ± 8.3 a	41.7 ± 16.0 a	50.0 ± 21.5 a
SPoS	0.0 ± 0.0 a	58.3 ± 16.0 a	41.7 ± 16.0 a
SPoS + FYM	8.3 ± 8.3 a	58.3 ± 16.0 a	33.3 ± 19.2 a
F value	0.600	1.653	0.929
df	3	3	3
*p* value	0.6310	0.2455	0.4655

* Pseudostem splitting scores of 0, 1 and 2, denote no splitting, mild splitting and severe splitting, respectively. Means followed by the same letter are not significantly different.

**Table 3:** Growth and yield parameters by banana cultivar and treatment. Means followed by the same letter are not significantly different (Tukey means separation; *p* > 0.05). Standard deviations are provided. ‘Nr’ denotes number.

Cultivar	Treatment	Nr of Plants Assessed for Flowering	Days to Flowering	Nr of Plants Assessed for Harvest	Days to Harvest	Bunch Weight (kg)	Nr of Clusters
Mpologoma	Control	57	475 ± 75 a	41	539 ± 53 a	19.2 ± 5.8 a	7.2 ± 1.0 a
	FYM	36	549 ± 52 ab	21	617 ± 42 b	15.4 ± 4.8 a	6.8 ± 0.8 a
	SPoS	48	483 ± 85 ab	35	539 ± 66 a	18.4 ± 4.9 a	7.2 ± 1.0 a
	SPoS + FYM	55	493 ± 88 ab	39	556 ± 72 ab	16.4 ± 6.1 a	6.8 ± 0.9 a
Ndizi	Control	8	558 ± 89 ab	2	578 ± 87 ab	18.5 ± 4.9 a	7.0 ± 0.0 a
	FYM	6	584 ± 52 b	1	580 ab	11.0 a	6.0 a
	SPoS	3	535 ± 70 ab	0	/	/	/
	SPoS + FYM	17	546 ± 84 ab	6	629 ± 53 b	12.1 ± 5.4 a	6.2 ± 1.5 a
F value			5.764		5.82	2.685	2.038
df			7		6	6	6
*p* value			<0.001		<0.001	0.017	0.0646

**Table 4:** Percentage of banana plants at different leaf symptom severity score thresholds at different days after planting and application of soil amendment SPoS (DAP). SPoS denotes spent *Pleurotus ostreatus* substrate. Controls had no external substrate. *n* = 4 plants nested within 3 rows. ‘NA’ denotes not applicable.

DAP	Treatments	% Plants (± SE) at Different Leaf Symptom Severity Score (0–4) Thresholds				
		* 0	1	2	3	4
163	Control	83.3 ± 8.3 a	8.3 ± 8.3 a	8.3 ± 8.3 a	0.00 ± 0.00	0.00 ± 0.00
	SPoS	91.7 ± 8.3 a	8.3 ± 8.3 a	0.00 ± 0.00 a	0.00 ± 0.00	0.00 ± 0.00
	F value	1.000	0.678	1.000	NA	NA
	*p* value	0.423	0.22	0.423	NA	NA
202	Control	83.3 ± 8.3 a	8.3 ± 8.3 a	8.3 ± 8.3 a	0.00 ± 0.00	0.00 ± 0.00
	SPoS	75.0 ± 14.4 a	25.0 ± 14.4 a	0.0 ± 0.0 a	0.00 ± 0.00	0.00 ± 0.00
	F value	0.142	1.000	1.000	NA	NA
	*p* value	0.742	0.423	0.423	NA	NA
218	Control	66.7 ± 33.3 a	25.0 ± 25.0 a	8.3 ± 8.3 a	0.00 ± 0.00	0.00 ± 0.00
	SPoS	75.0 ± 14.4 a	16.6 ± 8.3 a	8.3 ± 8.3 a	0.00 ± 0.00	0.00 ± 0.00
	F value	0.053	0.143	0.000	NA	NA
	*p* value	0.840	0.742	1.000	NA	NA
238	Control	50.0 ± 14.4 a	33.3 ± 22.0 a	16.7 ± 16.7 a	0.00 ± 0.00	0.00 ± 0.00
	SPoS	55.6 ± 5.6 a	44.4 ± 5.6 a	0.00 ± 0.00 a	0.00 ± 0.00	0.00 ± 0.00
	F value	0.082	0.165	1.000	NA	NA
	*p* value	0.802	0.724	0.422	NA	NA

* Leaf severity scores of 0, 1, 2, 3 and 4, respectively, represent no visual leaf symptoms, 1–33% of leaves turning yellow, 34–66% of leaves turning yellow with some old leaves hanging down the pseudostem, 67–100% of leaves turning yellow with some old leaves hanging down, and plant death with multiple brown leaves hanging down the pseudostem. Means followed by the same letter are not significantly different.

**Table 5:** Percentage of banana plants at different pseudostem splitting severity score thresholds (0 to 2) at different days after planting (DAP). SPoS denotes spent *Pleurotus ostreatus* substrate. Controls had no external substrate. *n* = 4 plants nested within 3 rows. Means followed by the same letter are not significantly different. ‘NA’ denotes not applicable.

DAP	Treatments	% of Plants (± SE) at Different Pseudostem Splitting Score (0–2) Thresholds		
		0	1	2
163	Control	100.0 ± 0.0	0.00 ± 0.00	0.00 ± 0.00
	SPoS	100.0 ± 0.0	0.00 ± 0.00	0.00 ± 0.00
	F	NA	NA	NA
	*p* value	NA	NA	NA
202	Control	100.0 ± 0.0 a	0.00 ± 0.00 b	0.00 ± 0.00
	SPoS	0.00 ± 0.00 b	100.0 ± 0.00 a	0.00 ± 0.00
	F	2.971 × 10^31^	2.971 × 10^31^	NA
	*p* value	<0.001	<0.001	NA
218	Control	83.3 ± 16.7 a	16.7 ± 16.7 b	0.00 ± 0.00
	SPoS	0.00 ± 0.00 b	100.0 ± 0.00 a	0.00 ± 0.00
	F	25.000	25.000	NA
	*p* value	0.038	0.038	NA
238	Control	100.0 ± 0.0 a	0.0 ± 0.0	0.0 ± 0.0 a
	SPoS	38.9 ± 20.0 a	0.0 ± 0.0	61.1 ± 20.0 a
	F	9.3078	NA	9.3078
	*p* value	0.0923	NA	0.0923

## Data Availability

The original contributions presented in this study are included in the article/Supplementary Material. Further inquiries can be directed to the corresponding author.

## Supplementary Materials

The following supporting information can be downloaded at: https://www.mdpi.com/article/10.3390/jof11110816/s1, Figure S1: Historical weather data (2010–2020) at Kawanda, Uganda; Figure S2: Percentage of plants at different corm damage score thresholds (i.e., 0 to 4), 687 days after treatment application. The treatments include a control without any amendment, farmyard manure (FYM), spent *P. ostreatus* substrate (SPoS) and a mixture of SPoS with FYM. The scores from 0 to 4, respectively, denote no internal corm symptoms, few internal spots to 1/3 discolored, 1/3–2/3 discolored, >2/3 discolored, and all inner corm sections (cortex to central cylinder) discolored. Means followed by the same letter are not significantly different at 5% Tukey HSD; Table S1: Average disease severity scores (of leaf and pseudostem) of the mother plants of the susceptible cultivar ‘Sukali Ndizi’ at each monitoring interval of experiment 1. FYM, and SPoS, respectively, denote farmyard manure and spent *Pleurotus ostreatus* substrate. Severity scores of treatments significantly different from the control plots are indicated in bold. Tukey means separation was performed at each time interval. At each time interval (days since planting), means followed by a different letter are significantly different (p-value indicated). At intervals when no significant differences (n.s.) were observed, letters are not indicated. Standard deviations are provided; Table S2: Average disease severity scores (of leaf and pseudostem) of the ratoon crop of the susceptible cultivar ‘Sukali Ndizi’ at each monitoring interval of experiment 1. FYM, and SPoS, respectively, denote farmyard manure and spent *Pleurotus ostreatus* substrate. Severity scores of treatments significantly different from the control plots are indicated in bold. Tukey means separation was performed at each time interval. At each time interval (days since planting), means followed by a different letter are significantly different (p-value indicated). At intervals when no significant differences (n.s.) were observed, letters are not indicated. Standard deviations are provided; Table S3: Average disease severity scores (of leaf and pseudostem) of the susceptible cultivar ‘Sukali Ndizi’ at each monitoring interval of experiment 2. SPoS denotes spent *Pleurotus ostreatus* substrate. Tukey means separation was performed at each time interval. At each time interval (days since planting), means followed by a different letter are significantly different (p-value indicated). At intervals when no significant differences (n.s.) were observed, letters are not indicated. Standard deviations are provided.
