# Supplementary material for: Field Evaluation of Spent Pleurotus ostreatus Substrate Reveals Limited Suppression of Fusarium Wilt in Banana
Source: J Fungi (Basel). 2025 Nov 18;11(11):816. doi: 10.3390/jof11110816 (PMC12653061; doi:10.3390/jof11110816)
Supplement: Supplementary file 1 [file jof-11-00816-s001.zip › jof-3981368-supplementary.pdf]

**Supplementary information**

Table S1: Average disease severity scores (of leaf and pseudostem) of the mother plants of the susceptible cultivar 'Sukali Ndizi' at each monitoring interval of Trial 1. Severity scores of treatments significantly different from the control plots are indicated in bold. Tukey means separation was performed at each time interval. At each time interval (days since planting), means followed by a different letter are significantly different (p-value indicated). At intervals when no significant difference (n.s.) was observed, letters are not indicated. Standard deviations are provided.

| **Day since planting** | **Treatment** | **Disease severity scores of** | | **P-values of scores** | |
|---|---|---|---|---|---|
| | | **Leaves** | **Pseudostem** | **Leaves** | **Pseudostem** |
| 224 | Control | 0.3 ± 0.5 | 0.1 ± 0.4 a | | |
| 224 | FYM | 0.1 ± 0.4 | 0.3 ± 0.6 ab | | |
| 224 | SPoS | 0.3 ± 0.6 | 0.3 ± 0.6 ab | | |
| 224 | SPoS + FYM | 0.1 ± 0.5 | **0.5 ± 0.7 b** | n.s. | P < 0.01 |
| 250 | Control | 0.6 ± 1.0 ab | 1.2 ± 0.9 | | |
| 250 | FYM | 0.3 ± 0.6 b | 1.2 ± 0.9 | | |
| 250 | SPoS | 0.8 ± 1.2 a | 1.1 ± 0.9 | | |
| 250 | SPoS + FYM | 0.3 ± 0.6 b | 1.2 ± 0.8 | P < 0.01 | n.s |
| 286 | Control | 0.3 ± 0.9 ab | 0.7 ± 0.6 a | | |
| 286 | FYM | 0.2 ± 0.8 a | 0.6 ± 0.5 ab | | |
| 286 | SPoS | 0.2 ± 0.8 a | 0.7 ± 0.5 a | | |
| 286 | SPoS + FYM | 0.7 ± 1.1 b | **0.4 ± 0.5 b** | P < 0.05 | P < 0.001 |
| 314 | Control | 0.1 ± 0.6 | 0.7 ± 0.7 | | |
| 314 | FYM | 0.1 ± 0.6 | 0.6 ± 0.5 | | |
| 314 | SPoS | 0.1 ± 0.4 | 0.6 ± 0.5 | | |
| 314 | SPoS + FYM | 0.1 ± 0.3 | 0.6 ± 0.5 | n.s | n.s |
| 346 | Control | 1.3 ± 1.5 | 1.0 ± 0.6 | | |
| 346 | FYM | 1.2 ± 1.4 | 1.0 ± 0.5 | | |
| 346 | SPoS | 1.3 ± 1.4 | 0.9 ± 0.6 | | |
| 346 | SPoS + FYM | 0.9 ± 1.3 | 0.9 ± 0.5 | n.s | n.s |
| 368 | Control | 1.2 ± 1.3 | 1.1 ± 0.5 a | | |
| 368 | FYM | 0.8 ± 1.3 | **0.8 ± 0.4 b** | | |
| 368 | SPoS | 0.8 ± 1.2 | 1.0 ± 0.2 ab | | |
| 368 | SPoS + FYM | 1.1 ± 1.3 | **0.9 ± 0.3 b** | n.s | P < 0.001 |
| 406 | Control | 1.3 ± 1.2 | 1.3 ± 0.6 a | | |
| 406 | FYM | 1.4 ± 1.5 | **0.9 ± 0.5 b** | | |
| 406 | SPoS | 1.3 ± 1.4 | **1.0 ± 0.2 b** | | |
| 406 | SPoS + FYM | 1.2 ± 1.3 | **0.7 ± 0.5 c** | n.s | P < 0.001 |
| 433 | Control | 1.4 ± 1.2 ab | 1.6 ± 0.6 | | |
| 433 | FYM | 1.6 ± 1.1 a | 1.4 ± 0.8 | | |
| 433 | SPoS | 1.1 ± 0.9 b | 1.4 ± 0.8 | | |
| 433 | SPoS + FYM | 1.2 ± 1.0 ab | 1.3 ± 0.8 | P < 0.05 | n.s |

Table S2: Average disease severity scores (of leaf and pseudostem) of the ratoon crop of the susceptible cultivar ‘Sukali Ndizi’ at each monitoring interval of Trial 1. Severity scores of treatments significantly different from the control plots are indicated in bold. Tukey means separation was performed at each time interval. At each time interval (days since planting), means followed by a different letter are significantly different (p-value indicated). At intervals when no significant difference (n.s.) was observed, letters are not indicated. Standard deviations are provided.

| **Day since planting** | **Treatment** | **Disease severity scores of** | | **P-values of scores** | |
|---|---|---|---|---|---|
| | | **Leaves** | **Pseudostem** | **Leaves** | **Pseudostem** |
| 470 | Control | 2.2 ± 0.5 | 1.4 ± 0.7 | | |
| 470 | FYM | 2.3 ± 0.5 | 1.6 ± 0.7 | | |
| 470 | SPoS | 2.0 ± 0.6 | 1.4 ± 0.8 | | |
| 470 | SPoS + FYM | 2.1 ± 0.8 | 1.1 ± 0.9 | n.s | n.s |
| 497 | Control | 2.2 ± 0.5 | 1.3 ± 0.5 | | |
| 497 | FYM | 2.2 ± 0.6 | 1.3 ± 0.7 | | |
| 497 | SPoS | 1.9 ± 0.3 | 1.0 ± 0.9 | | |
| 497 | SPoS + FYM | 2.2 ± 0.4 | 1.0 ± 0.9 | n.s | n.s |
| 530 | Control | 2.4 ± 0.7 | 1.4 ± 0.5 | | |
| 530 | FYM | 2.4 ± 0.7 | 1.2 ± 0.8 | | |
| 530 | SPoS | 2.0 ± 0.6 | 1.2 ± 0.6 | | |
| 530 | SPoS + FYM | 2.0 ± 0.0 | 1.3 ± 0.7 | n.s | n.s |
| 560 | Control | 3.0 ± 0.9 a | 1.6 ± 0.5 | | |
| 560 | FYM | 2.8 ± 0.6 a | 1.2 ± 0.6 | | |
| 560 | SPoS | 2.8 ± 0.4 a | 1.2 ± 0.5 | | |
| 560 | SPoS + FYM | **2.1 ± 0.3 b** | 1.3 ± 0.7 | P < 0.01 | n.s |
| 594 | Control | 2.7 ± 0.8 a | 1.5 ± 0.5 a | | |
| 594 | FYM | 2.8 ± 0.6 ab | 1.8 ± 0.5 a | | |
| 594 | SPoS | 2.6 ± 0.5 ab | 1.4 ± 0.5 a | | |
| 594 | SPoS + FYM | **2.1 ± 0.3 b** | **0.8 ± 0.4 b** | P < 0.05 | P < 0.001 |
| 615 | Control | 2.8 ± 0.9 a | 1.5 ± 0.5 | | |
| 615 | FYM | 2.2 ± 0.6 ab | 1.7 ± 0.5 | | |
| 615 | SPoS | 2.2 ± 0.5 ab | 1.3 ± 0.5 | | |
| 615 | SPoS + FYM | **1.8 ± 0.6 b** | 1.4 ± 0.5 | P < 0.01 | n.s |
| 651 | Control | 2.9 ± 0.7 a | 1.7 ± 0.5 | | |
| 651 | FYM | 2.6 ± 0.5 a | 1.6 ± 0.5 | | |
| 651 | SPoS | 2.6 ± 0.5 a | 1.6 ± 0.5 | | |
| 651 | SPoS + FYM | **1.9 ± 0.3 b** | 1.3 ± 0.5 | P < 0.001 | n.s |
| 687 | Control | 2.9 ± 0.9 a | 1.7 ± 0.5 | | |
| 687 | FYM | **2.2 ± 0.4 b** | 1.5 ± 0.5 | | |
| 687 | SPoS | 2.2 ± 0.9 ab | 1.4 ± 0.5 | | |
| 687 | SPoS + FYM | **1.9 ± 0.3 b** | 1.3 ± 0.5 | P < 0.01 | n.s |

Table S3: Average disease severity scores (of leaf and pseudostem) of the susceptible cultivar 'Sukali Ndizi' at each monitoring interval of Trial 2. Tukey means separation was performed at each time interval. At each time interval (days since planting), means followed by a different letter are significantly different (p-value indicated). At intervals when no significant difference (n.s.) was observed, letters are not indicated. Standard deviations are provided.

| **Day since planting** | **Treatment** | **Disease severity scores of** | | **P-values of scores** | |
|---|---|---|---|---|---|
| | | **Leaves** | **Pseudostem** | **Leaves** | **Pseudostem** |
| 163 | Control | 0.3 ± 0.6 | 0.0 ± 0.0 | | |
| 163 | SPoS | 0.1 ± 0.3 | 0.0 ± 0.0 | n.s. | n.s. |
| 202 | Control | 0.3 ± 0.6 | 0.0 ± 0.0 a | | |
| 202 | SPoS | 0.4 ± 0.9 | 1.0 ± 0.0 b | n.s. | P < 0.001 |
| 218 | Control | 0.0 ± 0.0 | 0.2 ± 0.4 a | | |
| 218 | SPoS | 0.3 ± 0.7 | 1.0 ± 0.0 b | n.s. | P < 0.001 |

Figure S1

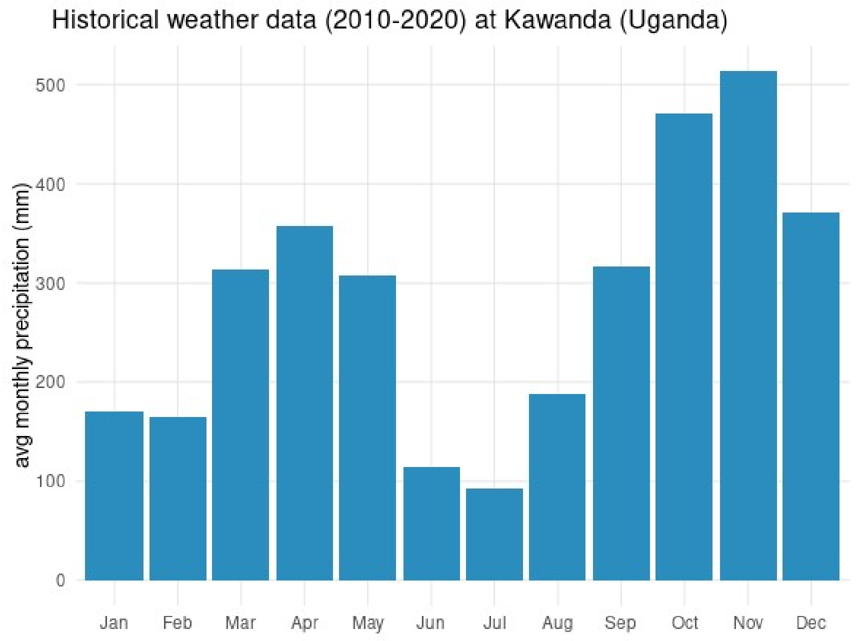


**Figure S1**: Historical weather data (2010-2020) at Kawanda, Uganda

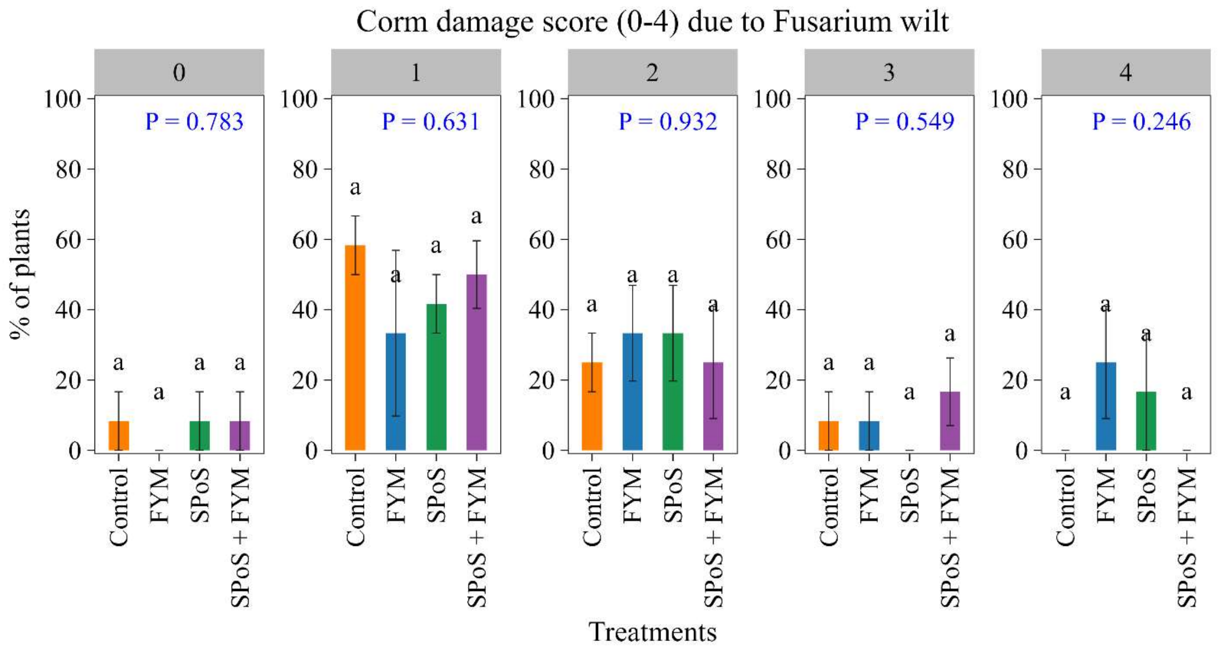


**Figure S2**. Percentage of plants at different corm damage score thresholds (i.e. 0 to 4), 687 days after treatment application. The treatments include a control without any amendment, farmyard manure (FYM), spent P. ostreatus substrate (SPoS) and a mixture of SPoS with FYM. The scores from 0 to 4, respectively, denote no internal corm symptoms, few internal spots to 1/3 discolored, 1/3-2/3 discolored, >2/3 discolored, and all inner corm sections (cortex to central cylinder) discolored. Means followed by the same letter are not significantly different at 5% Tukey HSD.
